# Molecular analysis of carnivore Protoparvovirus detected in white blood cells of naturally infected cats

**DOI:** 10.1186/s12917-018-1356-9

**Published:** 2018-02-05

**Authors:** Andrea Balboni, Francesca Bassi, Stefano De Arcangeli, Rosanna Zobba, Carla Dedola, Alberto Alberti, Mara Battilani

**Affiliations:** 1 Department of Veterinary Medical Sciences, Alma Mater Studiorum - University of Bologna, Via Tolara di Sopra 50, 40064 Ozzano dell’Emilia, BO Italy; 2 Department of Veterinary Medicine, University of Sassari, Via Vienna, 2, Sassari, 07100 Italy

**Keywords:** Canine parvovirus, Cat, Coinfection, Feline panleukopenia virus, White blood cells, PCR

## Abstract

**Background:**

Cats are susceptible to feline panleukopenia virus (FPV) and canine parvovirus (CPV) variants 2a, 2b and 2c. Detection of FPV and CPV variants in apparently healthy cats and their persistence in white blood cells (WBC) and other tissues when neutralising antibodies are simultaneously present, suggest that parvovirus may persist long-term in the tissues of cats post-infection without causing clinical signs. The aim of this study was to screen a population of 54 cats from Sardinia (Italy) for the presence of both FPV and CPV DNA within buffy coat samples using polymerase chain reaction (PCR). The DNA viral load, genetic diversity, phylogeny and antibody titres against parvoviruses were investigated in the positive cats.

**Results:**

Carnivore protoparvovirus 1 DNA was detected in nine cats (16.7%). Viral DNA was reassembled to FPV in four cats and to CPV (CPV-2b and 2c) in four cats; one subject showed an unusually high genetic complexity with mixed infection involving FPV and CPV-2c. Antibodies against parvovirus were detected in all subjects which tested positive to DNA parvoviruses.

**Conclusions:**

The identification of FPV and CPV DNA in the WBC of asymptomatic cats, despite the presence of specific antibodies against parvoviruses, and the high genetic heterogeneity detected in one sample, confirmed the relevant epidemiological role of cats in parvovirus infection.

## Background

Parvoviruses are non-enveloped single-stranded DNA viruses which infect a wide range of mammalian species, including several members of the order *Carnivora*. The Carnivore protoparvovirus 1, belonging to genus *Protoparvovirus*, family *Parvoviridae*, subfamily *Parvovirinae*, includes several closely related autonomous viruses causing a range of serious conditions, especially in young animals: feline panleukopenia virus (FPV, the prototype virus of the former carnivore parvovirus), canine parvovirus (CPV), mink enteritis virus (MEV), and raccoon parvovirus (RaPV) [1].

Feline panleukopenia virus has been known to be a cause of disease in cats since the beginning of the twentieth century, although there are other similar parvovirus species affecting cats, such as MEV and CPV. Natural infections in cats with CPV have been reported but FPV remains the most prevalent parvovirus causing disease in cats [2–4]. Since cats are susceptible to FPV and CPV 2a, 2b, 2c variants, superinfection and co-infection with multiple parvovirus strains associated with high viral genetic heterogeneity can occur with relatively high frequency in feline hosts [3, 5–7].

Parvoviruses commonly cause acute infection with high levels of viral shedding which generally ceases within 1–2 weeks post-infection, after the development of high titres of virus-neutralising antibody [8, 9]. Nevertheless, parvoviruses can be detected in faeces forup to 6 weeks after recovery, depending on the sensitivity of the diagnostic method used [10]. Cats experimentally infected with FPV shed the virus in both urine and faeces up to day 41–42 post-infection with parvovirus persisting in the lungs and kidneys for more than 50 weeks in cats which have recovered [11]. The detection of FPV and CPV variants in apparently healthy cats suggests that parvovirus infection may be common in some populations of clinically normal cats, and that asymptomatic cats may be able to shed parvovirus for prolonged periods of time [12–14]. Furthermore, the ability of FPV and CPV to persist in the peripheral blood mononuclear cells (PBMC) of cats irrespective of the presence of neutralising antibodies [13–17] and the presence of parvoviral DNA in the bone marrow of healthy cats [18], suggests that parvovirus may persist long term in the tissues of cats post-infection without causing clinical signs.

The aim of this study was to screen a population of 54 cats from Sardinia (Italy) for the presence of both FPV and CPV DNA within buffy coat samples. The DNA viral load, genetic diversity, phylogeny and antibody titres against parvoviruses were investigated in the cats testing positive to DNA parvoviruses.

## Methods

### Study design and sampling

This was a retrospective study, carried out on stored blood samples taken for routine diagnostic investigations undertaken at the Department of Veterinary Medicine, University of Sassari - UNISS (Sassari, NO Sardinia, Italy). Buffy coats from cats sampled between October 2011 and March 2012 were tested for the presence of feline and canine parvovirus DNA using real-time polymerase chain reaction (PCR). The partial VP2 gene of the viruses identified was sequenced and used for statistical analyses and phylogenetic comparisons. In addition, the sera of the cats which were positive to DNA parvoviruses were tested for the parvovirus antibody using the haemagglutination inhibition (HI) assay. Samples of owned or stray cats with different life-style conditions (indoor or outdoor, living alone or in community) were tested to evaluate different exposure to parvoviruses. Date of sampling, gender, age, breed, habitat and the clinical symptoms of the 54 cats sampled are reported in Table 1. According to these data, the animals were divided into two groups called A and B. The cats included in Groups A were predominantly healthy cats from four multi-cat households. The cats in Group B were sampled in the Emergency Room of the Veterinary Teaching Hospital of the Department of Veterinary Medicine (UNISS) and were predominantly stray cats showing different clinical signs (sick cats). Twenty-two of the 54 (40.7%) cats were clinically normal; 26/54 (48.1%) showed clinical signs attributable to various diseases; 3/54 (5.6%) showed gastrointestinal signs, such as vomiting and diarrhoea compatible with parvovirus infection; clinical status was unavailable for 3/54 (5.6%) cats. The vaccination status of the cats included in the study was unknown, although it was hypothesised that the stray cats were unvaccinated.

**Table 1:**
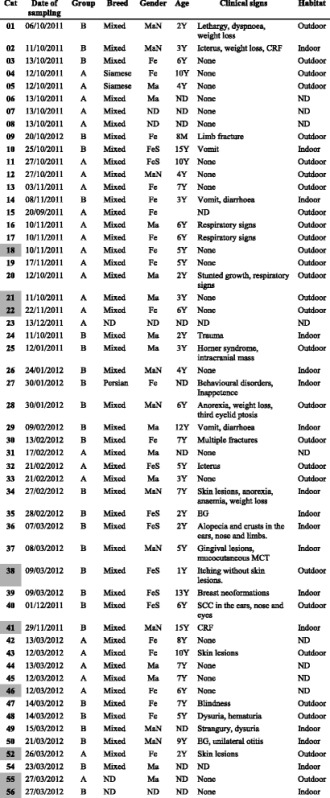
Characteristics of the 54 cats sampled

*Ma* male, *Fe* female, *MaN* male neutered, *FeS* female spayed, *Y* Years, *M* months, *CRF* chronic renal failure, *MCT* mast cell tumors, *SCC* Squamous cell carcinoma, *EG* Eosinophilic granuloma, *ND* Not determined

In grey: cats which tested positive for FPV or CPV

Anti-coagulated peripheral blood samples in ethylenediaminetetraacetic acid (EDTA) and coagulated blood for serology were collected from each cat. The blood samples were stored a + 4 °C and sera at − 20 °C until use.

### DNA extraction

Buffy coat-containing mononuclear cells was isolated from 3 ml of EDTA anti-coagulated peripheral blood samples using Histopaque-1077 (Sigma Aldrich, St. Louis, Mo, USA). The DNA was extracted using the DNeasy Blood and tissue Kit (QIAGEN, Hilden, Germany), according to the manufacturer’s instructions. The extracted DNA was eluted in 100 μl of ultrapure RNasi and DNasi free water, and was stored at − 20 °C after analysis.

### Detection of parvovirus infection using SYBR green real-time PCR

Parvovirus screening was carried out using real-time PCR using two conserved primers (A-for and B-rev, Table 2) targeting a 99 bp fragment of the VP2 gene. Quantitative PCR (qPCR) was carried out using SYBR Premix Ex Taq II (Takara Bio inc., Shiga, Japan) and the Rotor-Gene 3000 system (Corbett Research, Mortlake, NSW, Australia). The fluorescence signal was acquired on the FAM channel (multi-channel machine, source, 470 nm; detector, 510 nm; gain set to 5) with a fluorescence reading taken at the end of each elongation step. Each run consisted of an initial incubation in order to activate the hot-start DNA polymerase at 95 °C for 30 s followed by 40 cycles of denaturation at 95 °C for 10 s, annealing at 60 °C for 20 s and polymerisation at 72 °C for 30 s. During the melt cycle, the temperature was increased by increments of 1 °C from 65 °C to 95 °C. A pCR 4 plasmid (Invitrogen, Carlsbad, California, USA) containing one copy of the VP2 target sequence was produced as the external standard for the construction of the assay standard curve for quantitative analysis. Duplicates of six 10-fold dilutions of the standard plasmid, duplicates of the buffy coat DNA extracts of the cats sampled and a no template control were simultaneously analysed. Specimens were considered positive if the fluorescence curve in the amplification plot showed an exponential increase, and if a specific melting peak was observed. Copies of viral DNA were expressed per microlitre of DNA extract.

**Table 2:** Primers used

	Primer name	Primer sequence	Nucleotide position	Fragment amplified
Real Time-PCR	A-for	5’-AGCTACTATTATGAGACCAGCTGAG-3′	3767–3791	A-B: 99 bp
	B-rev	5′- CCTGCTGCAATAGGTGTTTTAA-3’	3844–3865	
Hemi-nested-PCR	C-for (P3^b^)	5’-CCATTTCTAAATTCTTTG −3’	3651–3668	C-D: 881 bp
	D-rev (VPrev^a^)	5’-TTTCTAGGTGCTAGTTGAG −3’	4513–4531	
	E-rev (P4^b^)	5’-AAGTCAGTATCAAATTCTT −3’	4201–4219	C-E: 569 bp

The primer positions refer to the nucleotide sequence of FPV reference strain CU-4 (GenBank accession number M38246). [41]^a^, [42]^b^

### Molecular characterisation of viral sequences

#### Amplification and sequencing of the VP2 gene

In order to differentiate between FPV and CPV variants 2a, 2b and 2c, a fragment of the VP2 gene coding for critical amino acid residues 297, 300, 305, 323 and 426 affecting the biological and antigenic proprieties was amplified and sequenced for each virus identified using real-time PCR. A hemi-nested PCR assay was developed by using three conserved primers (C-for, D-rev and E-rev, Table 2) for this purpose. Both PCR reactions were carried out using *Taq* DNA Polymerase (QIAGEN, Hilden, Germany) producing DNA fragments of 881 bp and 569 bp in length for the first and the second reaction, respectively. The temperature cycling protocol of the first amplification consisted of 94 °C for 5 min, 45 cycles with 1 cycle at 94 °C for 30 s, at 48 °C for 1 min, and at 72 °C for 1 min, followed by a final elongation at 72 °C for 10 min. In the second amplification, the PCR conditions were 94 °C for 5 min, 35 cycles with 1 cycle at 94 °C for 30 s, at 49 °C for 1 min, and at 72 °C for 45 s, followed by a final elongation at 72 °C for 10 min. In both PCR reactions, FPV 1033/09 [3] was used as a positive control while ultrapure water was used in each experiment to avoid false positive results. The nucleotide sequences were obtained using both forward and reverse primers.

Direct sequencing of the PCR products of one virus identified (number 41/2011) showed an unusually high number of ambiguities, suggesting a mixed viral population. Therefore, the amplification product was cloned into the pCR 4/TOPO vector using the TOPO cloning kit (Invitrogen, Carlsbad, CA, USA), and was transformed into *Escherichia coli* DH5α-competent cells according to the manufacturer’s protocol. Ten recombinant clones were sequenced using both forward and reverse primers.

#### Sequence data

The nucleotide sequences obtained were assembled and translated into amino acid sequences using BioEdit sequence alignment editor version 7.2.5 [19]. The VP2 assembled nucleotide sequences were aligned with reference sequences of feline and canine parvoviruses available in the GenBank (http://www.ncbi.nlm.nih.gov/genbank), including the modified live FPV vaccine strains available, using the ClustalW method implemented with BioEdit software.

A variety of statistical analyses aiming to investigate nucleotide diversity, sequence variability and natural selection were carried out on the sequence data set using DnaSP package version 5.10.01 [20]. Statistical analysis was carried out on the subpopulations, grouping the sequence data in the FPV, CPV and 41/2011 (all clones of sample 41/2011) clusters.

The following parameters were estimated for each cluster: total number of mutations (η), nucleotide diversity (π) and its standard error, total number of synonymous differences (SynDif), and total number of non-synonymous differences (NSynDif). Mutation frequency (total number of changes/total number of bases sequenced) and the percentage of mutated clones were used as indicators of genetic diversity of the viral population of virus 41/2011.

Phylogenetic relationships among the viruses detected and the parvovirus reference sequences were evaluated using MEGA version 7.0.20 [21]. The best-fit model of nucleotide substitution was determined using the Find Best DNA/Protein Model function implemented in MEGA, and the Tamura 3-parameter model was found to be optimal for all the sequence data (including reference strains). Phylogenetic trees were constructed using the Maximum Likelihood method, and bootstrap values were determined by 1000 replicates to assess the confidence level of each branch pattern.

#### Nucleotide sequence accession numbers

The nucleotide sequences obtained in this study have been submitted to the GenBank under accession numbers KT151621 to KT151638.

### Assessment of antibody titre by haemagglutination inhibition test

The parvovirus antibody titre was investigated using the haemagglutination inhibition (HI) test in the sera of the cats which tested positive to parvovirus DNA by direct molecular diagnosis. A CPV-2b isolate (number 115/2010), recovered from the faeces of a dog with non-fatal enteritis, and isolated by the authors on the Crandell Rees feline-kidney (CRFK, cell line was obtained from the Biobanking of Veterinary Resources of the Istituto Zooprofilattico Sperimentale della Lombardia e dell’Emilia Romagna IZSLER “Bruno Ubertini” http://www.ibvr.org/Services/CellCultures.aspx) cell line, was used as the viral antigen. This strain was used in HI experiments because there are no significant differences in serum titres inhibiting the haemagglutination reaction when cats are infected by FPV or CPV, if CPV is used as an antigen [22]. To calculate 8 haemagglutinating units (HAU) of the viral antigen, a haemagglutination (HA) assay was carried out on the CPV-2b isolate following procedures described previously by Carmichael et al. [22]. Porcine erythrocytes were washed three times in Bis-Tris Buffered Saline (BTBS, pH 6.2 at 4 °C) and suspended into 0.5% (*v*/v) BTBS containing 1% bovine serum albumin. To reveal the haemagglutination of both viruses, the test was conducted at a pH of 6–6.8 and was incubated at 4 °C [23, 24].

The HI tests were carried out as previously reported by Senda et al. [25]. All sera were tested in duplicate on two different plates; two-fold dilutions of a commercial canine hyperimmune serum raised against canine distemper, canine hepatitis and canine parvovirosis (Stagloban, ATI, Ozzano Emilia, BO, Italy), was used as positive controls.

For all sera, the mean value of the results from the duplicate HI tests was calculated. If the mean value of the result from one sera was included between two successive dilutions, the lower titre was reported. Cats with an HI titre ≥1:8 were considered positive and cats with an HI titre ≥1:80 were considered to be protected from infection.

## Results

### Detection of parvovirus in buffy-coat samples

The buffy coat DNA extracts of 54 cats were tested in duplicate for the presence of parvovirus using a SYBR Green real-time PCR assay which showed a limit of detection of 1 copy of the VP2 target DNA per microlitre of extract. Nine (16.7%) of the cats sampled tested positive: 18/2011, 21/2011, 22/2011, 38/2012, 41/2011, 46/2012, 52/2012, 55/2012 and 56/2012. Positive cats were detected in both groups A and B. Six positive cats were clinically normal (6 out of 22 clinically normal cats, 27.3%) and three showed clinical signs of various diseases (3 out of 26 cats with clinical signs of various diseases, 11.5%) (Table 1); no cat with gastrointestinal signs tested positive for parvovirus DNA. In the positive samples, the amount of viral DNA ranged from orders of magnitude 10^0^ to 10^2^ copies of DNA/μl of extract.

A melting curve analysis showed a solitary peak between 81.8° and 82 °C for standard plasmid dilutions and between 81° and 82 °C for each positive cat sample.

### Molecular characterisation of viral sequences

The nucleotide sequences of the partial VP2 gene obtained from positive samples were 532 bp in length (177 amino acid codons), and corresponded to residue 295–471 of the FPV reference strain CU-4 (GenBank accession number M38246). Nucleotide sequences of the VP2 partial gene were also obtained for ten recombinant clones of sample 41/2011, numbered from C01 to C10, respectively.

Analysis of the deduced amino acid residues at critical positions allowed the identification of the following carnivore protoparvoviruses: FPV (viruses 21/2011, 22/2011, 38/2012, 52/2012 and clones C01, C02, C03, C05, C06, C07, C08 and C10); CPV-2b (viruses 18/2011, 55/2012 and 56/2012) and CPV-2c (virus 46/2012 and clones C04 and C09). Six main viral populations were detected in cat 41/2011: clones C02, C05, C06, C07 and C10 resembled the predominant FPV circulating in the sampling area; clone C01 was an FPV which showed a change located in residue 312 (Lys → Arg); clone C03 was an FPV which displayed two coding changes in Thr391-to-Ala and Pro461-to-Ser; C04 was a CPV-2c with an amino acid change located at position 437 (Gly → Arg); C09 was a typical CPV-2c and clone 41/2011-C08 displayed amino acids identical to FPV in residues 297, 300, 305 and 323 while it showed glutamic acid typical of CPV-2c in position 426. The predicted amino acid sequence changes are summarised in Table 3.

**Table 3:**
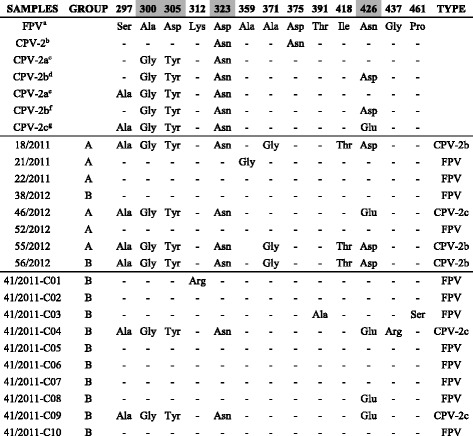
Change of amino acids to VP2 partial protein

The host-specific amino acids which differentiate FPV and CPV are represented by grey dashes

Deduced amino acid sequences of the VP2 gene were obtained from GenBank

^a^Prototype FPV: strain FPV-b (M38246)

^b^Prototype CPV type 2: strain CPV-b (M38245)

^c^Prototype CPV type 2a: strain CPV-15 (M24003)

^d^Prototype CPV type 2b: strain CPV-39 (M74849)

^e^Reference strain CPV type 2a: CPV-677 (AF306445)

^f^Reference strain CPV type 2b: CPV-637 (AF306450)

^g^Reference strain CPV type 2c: CPV-695 (AF01519)

Several synonymous and non-synonymous substitutions were detected by comparing the sequences as reported in Table 4. Feline panleukopenia viruses showed a total number of mutations slightly higher than CPV viruses, although synonymous changes predominated in the FPV sequences while non-synonymous changes were prevalent in the CPV viruses. Sample 41/2011 showed high genetic complexity generated within the host, and its sequence variability was higher than the variability of the other sequence data analysed as was seen by the values of parameter π. The non-synonymous fraction was greater for sample 41/2011, indicating clear prevalence of the number of non-synonymous mutations in the sample: 10 non-synonymous mutations out of a total of 15 mutations. The proportion of mutated viral clones was 60% for sample 41/2011 and the mutation frequency was on the order of 2.8 × 10^− 3^ (Table 4).

**Table 4:** Statistical analysis of the sequence data set

Sample	*η* ^b^	*π* ^c^	*SynDif* ^d^	*NSynDif* ^e^	% Mutated clones	Total mutations/bases sequenced	Mutation frequency
FPV (*n*^a^ = 4)	6	0.00627 (SE 0.00149)	5	1	˗	˗	˗
CPV (*n* = 4)	4	0.00376 (SE 0.00199)	1	3	˗	˗	˗
41/2011 (*n* = 10)	15	0.01032 (SE 0.00281)	5	10	60 (6/10)	15/5320	2.8 × 10^−3^

Summaries of sample sequence variability and quasispecies variation in the viral populations detected within cat 41/2011

^a^n: sample size

^b^*η*: total number of mutations

^c^*π*: nucleotide diversity

^d^*SynDif*: total number of synonymous differences

^e^*NSynDif*: total number of non-synonymous differences

**Fig. 1:**
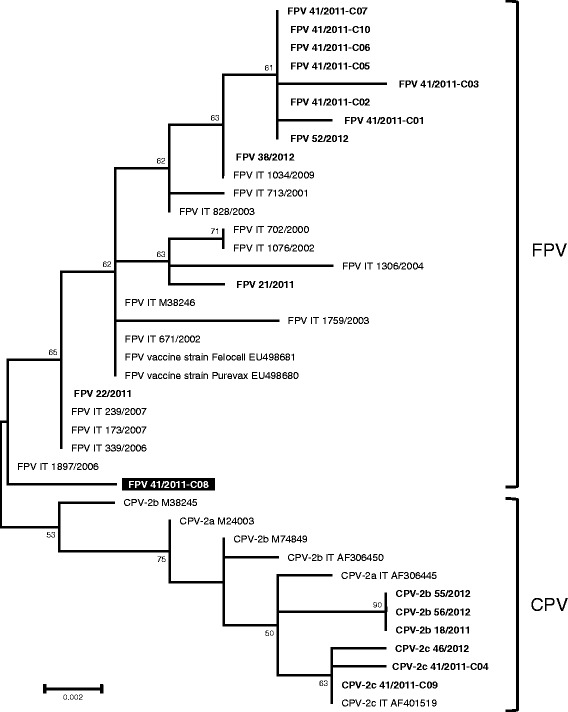
Unrooted phylogenetic tree. The phylogenetic tree was constructed with the nucleotide sequences of the partial VP2 gene generated in this study and with feline and canine parvovirus reference sequences available on the GenBank nucleotide database or analysed by the authors in a previous study (Battilani et al., [3]). Bootstrap values greater than 50%, calculated on 1000 replicates, are indicated on the respective branches. The feline and canine parvoviruses identified in Italy not detected in this study but included in the phylogenetic analysis are designated by IT. In bold: Nucleotide sequences generated in this study. Underlined: clone 41/2011-C8

Unrooted phylogenetic trees constructed from alignments of the nucleotide sequences obtained in this study with additional reference sequences showed two main clusters referable to FPV and CPV. The sequence of clone 41/2011-C08 formed a monophyletic branch inside the FPV clade (Fig. 1). Nucleotide sequences of the modified live FPV vaccine strains available from GenBank were distinguishable from all the FPV and CPV sequences obtained in this study, and direct relationships between them were not evident from the phylogenetic tree.

### Haemagglutination inhibition test

The HI titres obtained are reported in Table 5. All the cats which tested positive for parvovirus DNA were also positive for antibodies against parvoviruses; cats 22/2011 and 55/2012 showed a titre higher than 1:80.

**Table 5:** The HI titre of cats which were positive in Real-time PCR

Sample	HI titre
18/2011	1:32
21/2011	1:32
22/2011	1:256
38/2012	1:16
41/2011	1:64
46/2012	1:32
52/2012	1:32
55/2012	1:256
56/2012	1:16
Stagloban	1:256

## Discussion

The present study screened the occurrence of parvovirus DNA and the feline host-immune status in a cat population. Asymptomatic and symptomatic cats sampled in Sardinia (Italy) during 2011–2012 were tested for the presence of both FPV and CPV DNA in WBC using qPCR, and the partial VP2 gene of the viruses identified was characterised. Parvoviruses have commonly been titrated in the faeces of infected animals, insofar as the viruses shed in faeces reflect virus replication; the Authors chose to analyse the WBC for the presence of viral DNA as, in addition to the intestinal crypt cells, bone marrow and other lymphoid tissues are also a major target for parvoviruses in both dogs and cats [9]. Furthermore, since parvovirus can frequently be isolated in infected cats, even in the presence of high virus-neutralising antibodies [15, 17], antibody titres against parvovirus were established in the sera of positive cats using an HI assay.

Nine (16.7%) out of a total of 54 cats tested positive for parvovirus DNA with viral DNA quantities ranging from 10^0^ to 10^2^ copies/μl of extract, demonstrating that the viral genome was detectable, although at low levels, in the WBC of a relatively large number of cats. All the cats which tested positive for parvovirus DNA had antibodies against parvoviruses. Positive cats included healthy and diseased cats. Although sick cats had a potentially greater probability of being persistently infected, none of the samples from cats showing signs of gastroenteritis tested positive.

The presence of FPV and CPV DNA in the faecal and peripheral blood samples of healthy cats has previously been reported [3, 12, 15–17], raising important questions regarding the role of cats in the epidemiology of parvoviruses. In our study, the absence of clinical signs consistent with parvovirosis in the cats which tested positive, together with the low amount of viral DNA detected, suggested that the infection was asymptomatic or that residual viral DNA remains in the organism after recovery from acute infection. Furthermore, the detection of parvoviral DNA in WBC reveals the presence of the virus in the bone marrow or in other lymphoid tissues which might reflect chronic or latent infection. The detection of CPV DNA in apparently healthy domestic cats confirmed a previous survey in which a high prevalence (37%) of CPV in apparently healthy domestic cats living in rescue shelters was identified [14]. Canine parvovirus-like DNA was also detected in the tissues of wildlife carnivores which had no clinical signs of active infection and, therefore, it was likely that this virus caused latent or persistent infection not only in domestic cats [26]. Animal parvoviruses, such as Rodent protoparvovirus and Aleutian mink disease parvovirus, have been shown to persist in their host [27, 28]. Persistence is a common feature also for human parvovirus B19 (B19V) infection [29]: B19V parvoviral DNA has been documented in a wide range of tissues and the bone marrow of asymptomatic adults, although the majority of people harbour parvoviral DNA in a form which does not actively replicate [30, 31].

Of the nine cats testing positive for parvovirus DNA, four showed FPV DNA, four CPV DNA (three CPV-2b and one CPV-2c), and cat 41/2011 showed unusually high genetic diversity and evidenced DNA belonging to two species of parvovirus in the same patient.

The pathogenicity of CPV variants for cats is not fully understood. Some studies have suggested that CPV had the same pathogenic potential as FPV in cats [3, 32–35]; in other studies, clinical signs were not observed in infected animals, with the exception of transient leukopenia [36, 37]. These results led to the speculation that CPV, compared to FPV, can most frequently cause asymptomatic and persistent infection in cats, even if additional studies are clearly needed to fully understand the potential of cats as CPV carriers. In our study, an equivalent prevalence of FPV and CPV was found in the samples examined; this result might be related to the type of cat population sampled, which consisted mainly of healthy cats and cats showing clinical signs not related to parvovirosis. Alternatively, it could be due to the biological matrix analysed since parvoviruses have commonly been investigated in the faeces of infected animals; instead, in our survey the WBC were analysed.

The rates of variation of the FPV and CPV nucleotide sequences analysed in this study were similar, although genetic diversity in the FPV sequences was generated primarily by synonymous mutations which did not result in amino acid substitutions. This result was congruent with the evolutive behaviour of FPV which, since its emergence in 1920, has not undergone significant changes in antigenic and biological properties. Feline panleukopenia virus varied at a slow rate by random genetic drift and it maintained host-specificity [38]. Instead, in the CPV sequence data set, non-synonymous mutations were predominant. This finding is compatible with the pattern of evolution observed for CPV. Since its emergence in the late 1970s, CPV evolution has been driven by strong positive selection, giving rise to new antigenic variants which have replaced the original type [38].

An unusual genetic complexity was reported for sample 41/2011, with six different viral DNAs ascribable to two distinct species of parvovirus, FPV and CPV type 2c. Although co-infection by more than one parvovirus species is a rare event, it has already been described in a cat simultaneously infected by FPV and CPV-2a [7].

Carnivore protoparvoviruses show an estimated annual substitution rate on the order of 10^− 4^ to 10^− 5^ whereas the mutation frequency detected in sample 41/2011 was on the order of 2.8 × 10^− 3^, a value which determines the quasispecies distribution in RNA virus populations. Carnivore parvoviruses are very prone to genetic evolution, showing substitution rates similar to those of RNA viruses, with values of approximately 10^− 4^ substitutions per site per year. This result, together with the detection of CPV-2c, which has already been reported in multiple infections of high genetic complexity [3, 5, 39], confirmed that co-infection with different species of parvovirus in feline hosts led to a high genetic variability and to the potential emergence of new viruses [3].

The presence of distinctive mutations between the FPV DNA sequences detected and the sequences of modified live FPV vaccine strains available, together with the lack of information regarding the vaccination history of the cats sampled, allowed the Authors to exclude the possibility that DNA from vaccine strains was detected and did not allow speculation regarding the persistence of modified live vaccine strains in the cats sampled. However, viraemia persisting up to 24 days post vaccination has been reported in dogs vaccinated with modified live canine parvovirus [40].

## Conclusions

Additional studies are required to investigate the nature and clinical significance of the presence of parvovirus DNA in the WBC of healthy cats and the potential of cats as parvovirus carriers. Ideally, faeces for the examination of shedding should also be included in the sampling; viral isolation could be useful for testing viral vitality, and reverse transcription-PCR assay targeting viral mRNA could be carried out to determine if there is active replication.

## Abbreviations

BTBS: Bis-Tris Buffered Saline
CPV: Canine parvovirus
CRFK: Crandell feline kidney
EDTA: Ethylenediaminetetraacetic acid
FPV: Feline panleukopenia virus
HA: Haemagglutination assay
HAU: Haemagglutinating units
HI: Haemagglutination inhibition
MEV: Mink enteritis virus
PCR: Polymerase chain reaction
qPCR: Quantitative PCR
RaPV: Raccoon parvovirus
TAE: Tris-acetate-EDTA
VP2: Viral protein 2
WBC: Whole blood cells
